# DeepHLAPred: a deep learning-based method for non-classical HLA binder prediction

**DOI:** 10.1186/s12864-023-09796-2

**Published:** 2023-11-23

**Authors:** Guohua Huang, Xingyu Tang, Peijie Zheng

**Affiliations:** 1 School of Information Technology and Administration, Hunan University of Finance and Economics, Changsha, Hunan 410215 China; 2 College of Information Science and Engineering, Shaoyang University, Shaoyang, Hunan 422000 China

**Keywords:** Non-classical HLA class I, Deep learning, Representation, Information entropy, Convolutional neural network

## Abstract

Human leukocyte antigen (HLA) is closely involved in regulating the human immune system. Despite great advance in detecting classical HLA Class I binders, there are few methods or toolkits for recognizing non-classical HLA Class I binders. To fill in this gap, we have developed a deep learning-based tool called DeepHLAPred. The DeepHLAPred used electron-ion interaction pseudo potential, integer numerical mapping and accumulated amino acid frequency as initial representation of non-classical HLA binder sequence. The deep learning module was used to further refine high-level representations. The deep learning module comprised two parallel convolutional neural networks, each followed by maximum pooling layer, dropout layer, and bi-directional long short-term memory network. The experimental results showed that the DeepHLAPred reached the state-of-the-art performanceson the cross-validation test and the independent test. The extensive test demonstrated the rationality of the DeepHLAPred. We further analyzed sequence pattern of non-classical HLA class I binders by information entropy. The information entropy of non-classical HLA binder sequence implied sequence pattern to a certain extent. In addition, we have developed a user-friendly webserver for convenient use, which is available at http://www.biolscience.cn/DeepHLApred/. The tool and the analysis is helpful to detect non-classical HLA Class I binder. The source code and data is available at https://github.com/tangxingyu0/DeepHLApred.

**Supplementary Information:**

The online version contains supplementary material available at 10.1186/s12864-023-09796-2.

## Introduction

Human leukocyte antigen (HLA) genes are located at the human histocompatibility complex (MHC) region on the short arm of chromosome 6 [1, 2]. HLA genes have more than one different allele, which are encoded into cell-surface glycoproteins which play a key role in the immune system [3, 4]. Generally, HLA genes are classified into three categories, class I, class II, and class III [5], while HLA class I genes are further divided into two subcategories: classical (HLA-A, HLA-B, HLA-C) and non-classical (HLA-E, HLA-G, HLA-F) [6]. As of Feb 2023, the IPD-IMGT/HLA database deposited 25,228 HLA Class I alleles, including 7712 HLA-A, 9164 HLA-B, 7672 HLA-C, and 10,592 HLA Class II alleles [7, 8]. The non-classical HLA class I genes are different from classical I ones in a wide range of respects including specific patterns of transcription, protein expression, and immunological functions [9]. For example, non-classical HLA class I genes are less polymorphic than classical, characterized by a low genetic diversity and by a particular expression pattern, structural organization, and functional profile [10–13].

An adaptive immune response was activated by binding of peptides from antigenic pathogens to HLA and then eliminated the source pathogens [14]. Therefore, identifying the HLA binding peptides not only helps understand the immune mechanism, but also facilitates rational subunit vaccine design. However, this is still a bottleneck to precisely recognize the non-classical HLA binders at present [15]. Hannoun et al. employed the biochemical methodology to identify 4 HIV-1-derived HLA-E-binding peptides in assays [16]. This methodology is very complex, time-consuming, and laborious [17]. Over the recent twenty years, computational methods have attracted more attention due to simplicity and effectiveness. No less than ten computational methods have been proposed for predicting HLA binders [15, 18–25].

In 1993, Bisset et al. employed the neural network to determine HLA-DR1 binding peptides [18]. Trained by the peptide segments known to bind to HLA-DR1, the neural network was able to learn representations relating to HLA-DR1-binding capacity to a certain extent. Singh et al. developed a graphical web tool to identify HLA-DR binder [15] and an online web tool to predict peptides binding to MHC class-I alleles [19]. Nielsen et al. utilized the stabilization matrix method to develop a quantitative MHC class II binding prediction [26]. Lata et al. created a support vector machine-based method for prediction of promiscuous binders of MHC class II alleles [27]. Wang et al. combined multiple machine learning algorithms to explore HLA-peptide binding affinities for HLA DR, DP, and DQ alleles [28]. Peters et al. set up a benchmark dataset for detecting peptide binding to MHC-I alleles, and compared the neural network-based and two matrix-based predictions [29]. Lin et al. compared and evaluated thirty prediction servers for seven human MHC-I molecules and argued that non-linear predictors were superior to matrix-based ones [30]. Nielsen et al. developed a pan-specific HLA-DR prediction [31], while Jurtz et al. fused the eluted ligand and peptide binding affinity data to promote prediction of peptide-MHC class I interaction [20]. Most of computational methods above were based on the traditional machine learning (shallow learning), which were restricted to the small number of leaning samples. The generalization ability of the model was sometimes not as good as expected. Ye et al. [22] employed long short-term memory (LSTM) and multiple head attentions to build a deep learning-based method (MATHLA) for classical HLA class I binding peptide prediction. The MATHLA showed the improved accuracy of prediction for HLA-C alleles and depicted some HLA-ligand binding patterns [22]. Zhang et al. proposed a complex model (HLAB) for HLA class I binding peptide prediction [23]. The HLAB used the pre-trained Protein Bidirectional Encoder Representations (ProBERT) [32] to extract initial representations from peptides,, which is a BERT model [33–35] trained by the protein sequences from the UniRef100 [36] as well as BFD [37] databases then employed bi-directional LSTM (Bi-LSTM) to refine contextual semantics, utilized the Umap [38] to reduce the dimensions, and finally built seven binary classification models. Chu et al. [24] proposed a transformer-based method for peptide-HLA binding prediction. The experiments showed superior performance over 14 state of art methods.

More attentions were paid to classical HLA genes than non-classical HLA class I genes in the past ten years [39]. However, the recent studies have demonstrated that non-classical HLA class I alleles play equally important roles in transcription, protein expression, and immune regulation [9, 13, 40–45]. To best of our knowledge, only the HLAncPred [6] was explicitly intended to predict binders for non-classical HLA class I alleles. The HLAncPred was a feature engineering and traditional machine learning-based method, which used different machine learning algorithms with different representations to construct the predicting models. Although the HLAncPred obtained the quite high performance, it was inconvenient to choose a specified model for multiple-type datasets. Hence, it is necessary to develop a more efficient method for non-classical HLA binder prediction. Here, we developed a deep learning-based method for non-classical HLA binder prediction, called DeepHLAPred. The DeepHLAPred first extracted initial representations of non-classical HLA binding and non-binding peptide sequences by three encoding methods, and then fed them into an embedding layer followed by a deep leaning module which consisted of two parallel sequences. Each sequence comprised mainly convolutional neural network (CNN) at different scale and Bi-LSTM. The two fully connected layers were attached to the deep leaning module for the decision. To validate the effectiveness and efficiency of the DeepHLAPred, we tested it extensively on the balanced, the unbalanced, and the independent datasets.

## Materials and methods

### Materials

Adequate and reliable data is crucial for building a robust predictive model. We used the non-classical class I HLA binding peptides collected by Dhall et al. [6] as the benchmark datasets. All the binding peptides were experimentally validated by the fluorescence-based, and the mass spectrometry or the X-ray crystallography, which were of 8 to 15 amino acid residues. Dhall et al. [6] grouped the peptides into two categories: the balanced and the imbalanced, each with five datasets. In the balanced category, each dataset included the equal numbers of the positive and the negative samples, while the number of the negative samples was ten times more than the number of positive ones for each dataset in the imbalanced category. The positive samples were identical for both the balanced and the imbalanced category. The binding peptides (positive samples) for HLA-E∗01:01, HLA-E ∗01:03, HLA-G∗01:01, HLA-G∗01:03, and HLA-G∗01:04 alleles were 142, 632, 2633, 751, and 812, respectively. Peptides of all the binders were downloaded from the website: https://webs.iiitd.edu.in/raghava/hlancpred.

### DeepHLAPred framework

**Fig. 1:**
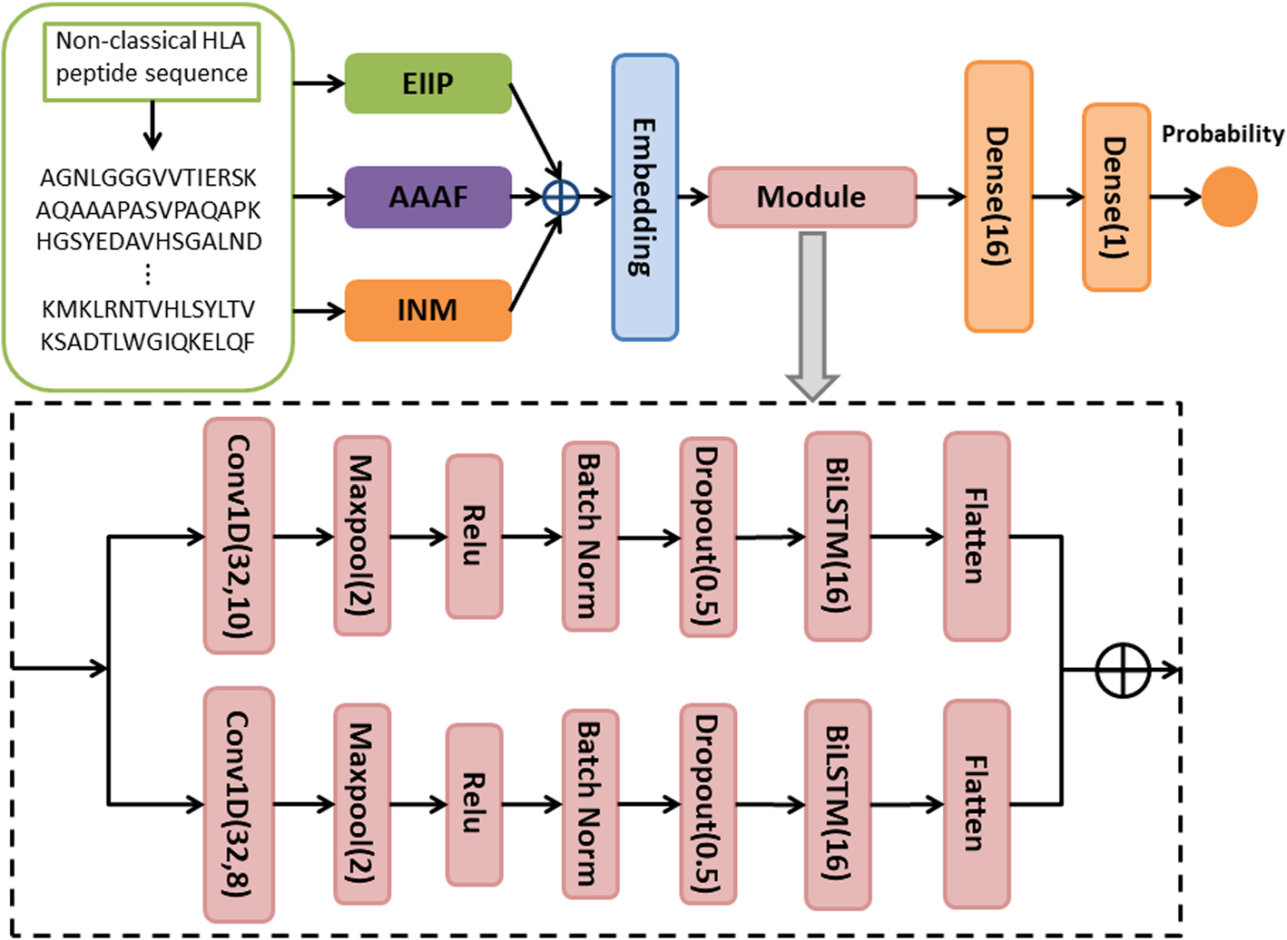
The flowchart of DeepHLAPred. Dense stands for fully-connected layer. The numbers in the bracket represent value of corresponding parameters

Figure 1 showed the schematic framework of DeepHLAPred. The binding peptides were first encoded by electron-ion interaction pseudo potential (EIIP), integer numerical mapping (INM), and accumulated amino acid frequency (AAAF), which then passed through the embedding layer. Two parallel CNNs were employed to further refine high-level abstract information, each followed by max pooling, by Batch Normalization, by Dropout, and by Bi-LSTM. The Bi-LSTM was intended to learn the dependency relationship in the peptides. Lastly, the fully connected layer was attached to the Bi-LSTM layer. The sigmoid activation function was used for decision in the last fully connected layer, which outputted a probability value between 0 and 1. If the probability value was greater than 0.5, it was determined as non-classical HLA class I binders, and otherwise it was non-classical HLA non-binders. The detailed model parameters were shown in the Supplementary Table 1. The formula of the sigmoid function was expressed as:

1 $$\begin{array}{c}Sigmoid\left(x\right)={\left(1+{e}^{-x}\right)}^{-1}\end{array}$$

#### EIIP

The EIIP was defined as the energy of delocalized electrons of amino acid [46], which is one of the most important physical property of amino acid. We used the EIIP to encode each amino acid (Table 1). For example, the peptide sequence “CEFSQC” was encoded by the EIIP into (0.08292, 0.00580, 0.09460, 0.08292, 0.07606, 0.08292). The EIIP of a peptide reflected the distribution of the free electron energies.

**Table 1:** The EIIP and INM value of each amino acid

Amino Acid	EIIP	INM	Amino Acid	EIIP	INM
Alanine(A)	0.37100	1	Leucine(L)	0.00000	11
Arginine(R)	0.95930	2	Lysine(K)	0.37100	12
Asparagine(N)	0.00359	3	Methionine(M)	0.08226	13
Asparticacid(D)	0.12630	4	Phenylalanine(F)	0.09460	14
Cystine(C)	0.08292	5	Proline(P)	0.01979	15
Glutarnine(Q)	0.07606	6	Serine(S)	0.08292	16
Glutamicacid(E)	0.00580	7	Threonine(T)	0.09408	17
Glycine(G)	0.00499	8	Tryptophan(W)	0.05481	18
Histidine(H)	0.02415	9	Tyrosine(Y)	0.05159	19
Isoleucine(I)	0.00000	10	Valine(V)	0.00569	20

#### INM

In order to solve the problem of sparse dimension caused by one-hot encoding, we assigned different positive integer values to twenty amino acids (Table 1). We used MathFeature [47] to compute the INM. The MathFeature is a python package which is able to compute up to 37 categories of representations for DNA, RNA or protein sequences. For example, the sequence “CEFSQC” was mapped into a numeric vector (5, 7, 14, 16, 6, 5).

#### AAAF

The AAAF [47] reflected the distribution density of amino acid in a protein sequence. Assuming a non-classical HLA Class I binding peptide sequence $\text{S}={s}_{1}{s}_{2}\cdots {s}_{n}$, where $n$ denoted the length of the sequence S. The AAAF was computed by

2 $$\begin{array}{c}f\left({s}_{j}\right)=\frac{1}{j}\sum\limits _{t=1}^{j}T\left({s}_{t}\right)\end{array}$$

3 $$\begin{array}{c}T\left(s_t\right)=\end{array}\left\{\begin{array}{cc}1,&s_t=s_j\\0,&s_t\neq s_j\end{array}\right.$$

A peptide sequence of $n$ residues was of $n$ dimensional AAAF feature. For example, the AAAF of the sequence “CEFSQC” was (1.0000, 0.50000, 0.33333, 0.25000, 0.20000, 0.33333). We also used the MathFeature [47] to compute the AAAF.

#### CNN

The CNN is a feed-forward neural network [48, 49] that is one of the most popular algorithms in the area of deep learning. It significantly reduces the number of training parameters [48, 50]. The CNN consists mainly of convolutional and pooling operation. The convolutional operation is called also the filter operation. In order to refine multiple-view representations, the CNN uses more than a filter (kernel). The pooling operation is a down-sampling technique, which reduces computations and overfitting to a certain extent. Compared with traditional neural networks, the CNN is characterized by weight sharing and local connectivity. Over the past decades, CNN has achieved remarkable success in various fields, such as medical image analysis [51, 52], speech recognition [53], target detection [54], natural language processing [55–58]. We applied two parallel one-dimensional convolutional operations which are of different scale. One was with the kernel size of 10 and another was with the kernel size of 8. The max pooling operation with a pooling window size of 2 was attached to the corresponding convolution. RELU was used as the activation function. The batch normalization and the dropout were used to reduce overfitting. The dropout rate was set to 0.5.

#### Bi-LSTM

The LSTM is actually a kind of recurrent neural network (RNN), which is of gate mechanism [59–61]. Each repeated module in the common LSTM consists of the input gate, the output gate, forget gate and the cell state. At the heart of LSTM is the cell state, which preserves previous record. The forget gate determines what information of previous state cell is forgot or remembered. The input gate determines what new information is added to the cell state. The candidate value is created by the tanh function. The forget gate, the candidate value and the input gate jointly update the cell state. The hidden state is updated by the output gate and the cell state. The LSTM well solve the long-term dependence, gradient vanishing, or gradient exploding problems [62–64]. The Bi-LSTM captures bidirectional relationship between words (token). In this study, we used the Bi-LSTM.

## Model evaluation

We used the following five evaluation metrics: SN(sensitivity), SP(specificity), $\text{A}\text{C}\text{C}$ (accuracy), $\text{M}\text{C}\text{C}$ (Matthews correlation coefficient) to measure the performance [65, 66]. Their formulas were expressed as:

4 $$\begin{array}{c}SN{}_{}=\frac{{T}_{P}}{{T}_{P}+{F}_{N}}\end{array}$$

5 $$\begin{array}{c}SP{}_{}=\frac{{T}_{N}}{{T}_{N}+{F}_{P}}\end{array}$$

6 $$\begin{array}{c}ACC=\frac{{T}_{P}+{T}_{N}}{{T}_{P}+{T}_{N}+{F}_{P}+{F}_{N}}\end{array}$$

7 $$\begin{array}{c}MCC=\frac{{T}_{P}\times {T}_{N}-{F}_{P}\times {F}_{N}}{\sqrt{\left({T}_{P}+{F}_{N}\right)\left({T}_{P}+{F}_{P}\right)\left({T}_{N}+{F}_{P}\right)\left({T}_{N}+{F}_{N}\right)}}\end{array}$$

In addition, we used ROC curves (receiver operating characteristic curves) to visualize the performance. The ROC curve is to link true positive rate (TPR) against false positive rate (FPR) under various threshold. TPR and FPR were defined by

8 $$\begin{array}{c}TPR=\frac{{T}_{P}}{{T}_{P}+{F}_{N}}\end{array}$$

9 $$\begin{array}{c}FPR=\frac{{F}_{P}}{{F}_{P}+{T}_{N}}\end{array}$$

The area under the ROC curve (AUC) was employed to quantitatively assess performance. In the above equations, ${\text{T}}_{\text{P}}$, ${\text{T}}_{\text{N}}$, ${\text{F}}_{\text{P}},$ and ${\text{F}}_{\text{N}}$ were denoted as true positive (number of samples correctly as positive), true negative (number of samples correctly predicted as negative), false positive (number of samples incorrectly predicted as positive), and false negative (number of samples incorrectly predicted as negative), respectively.

## Results and discussions

### Cross validation on the balanced category

**Fig. 2:**
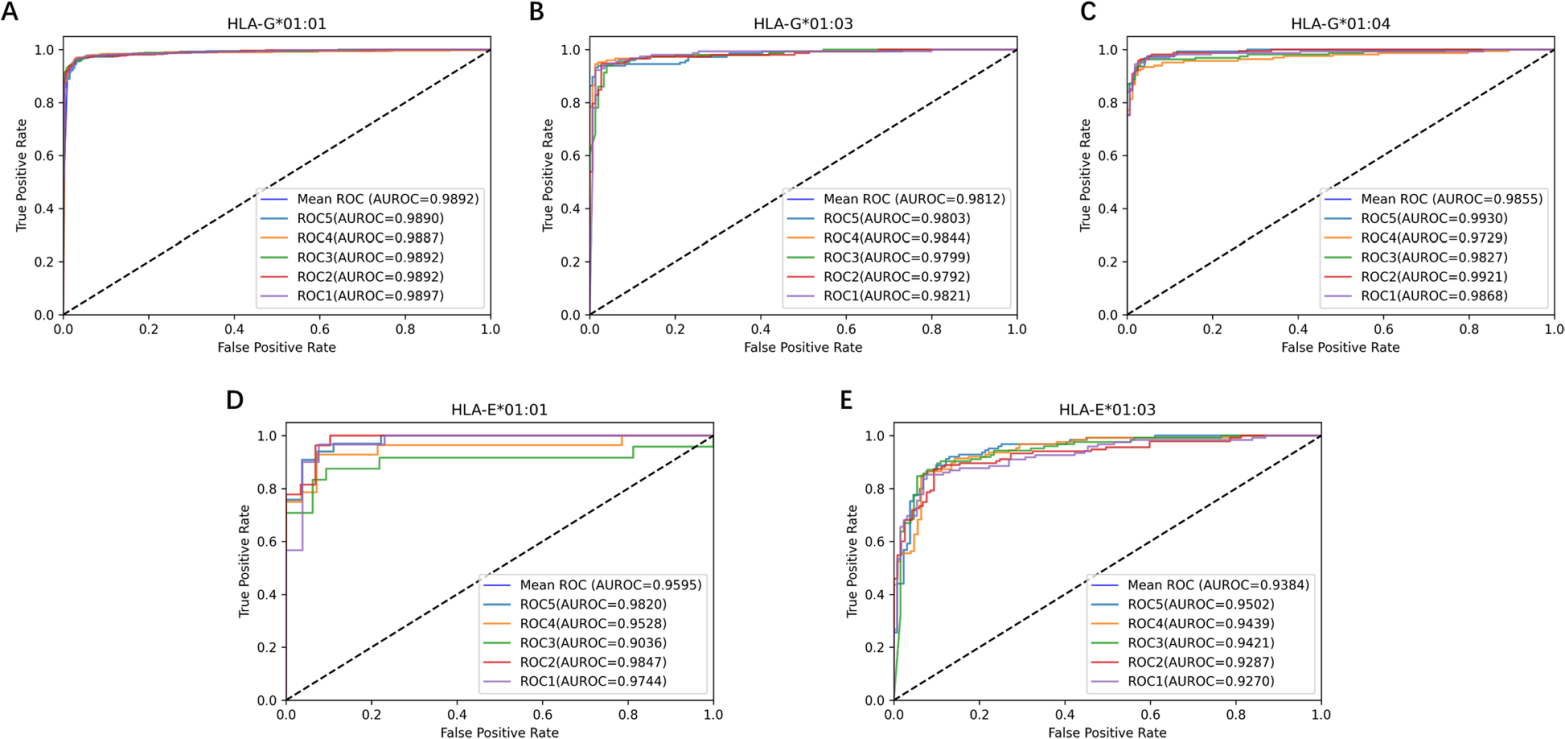
The ROC curves and AUC values on the five-fold cross validation

**Fig. 3:**
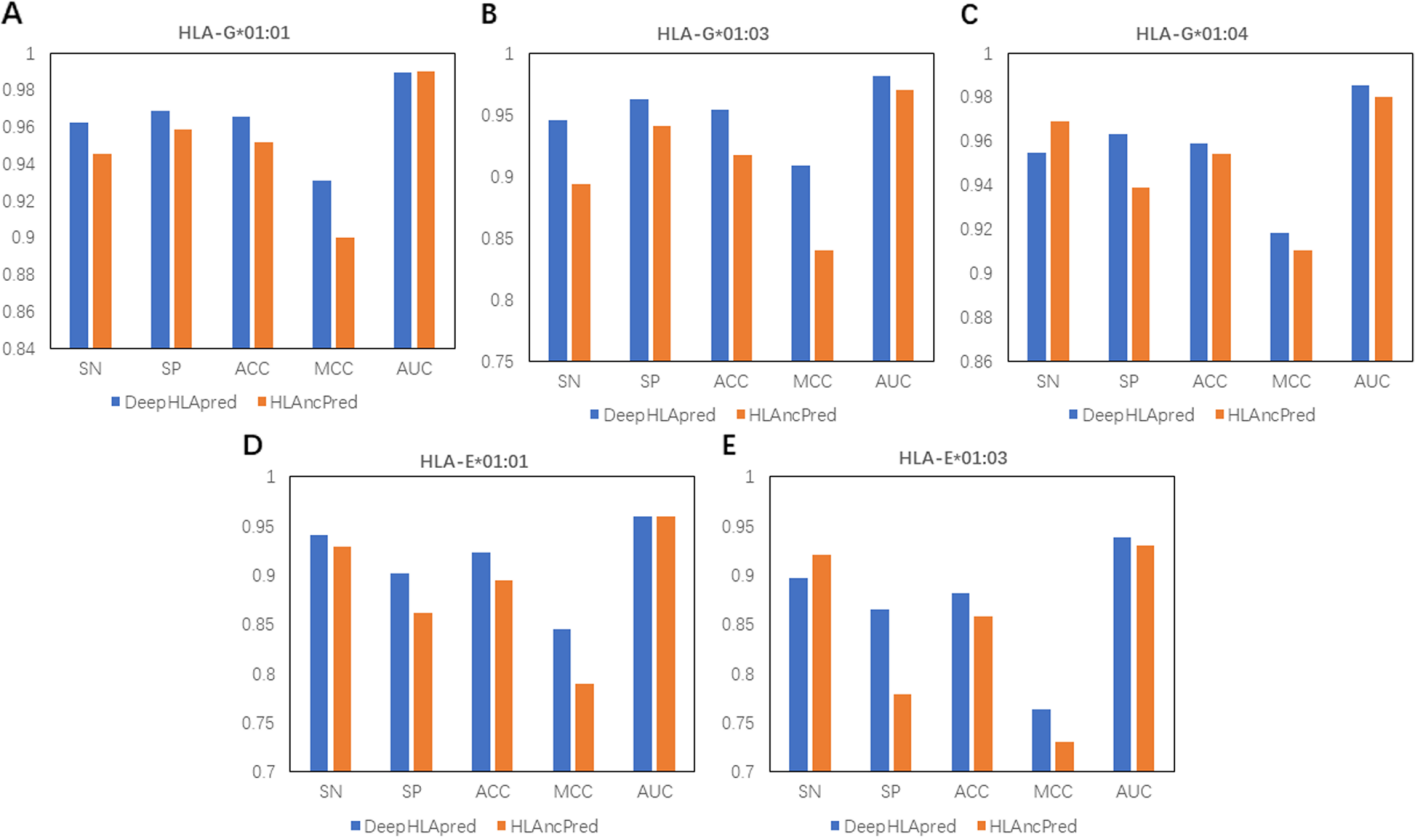
Comparison with state-of-the-art methods on five-fold cross-validation in balanced datasets

We conducted five-fold cross-validation on five balanced datasets (HLA-G*01:01, HLA-G*01:03, HLA-G*01:04, HLA-E*01:01, HLA-E*01:03) to examine the DeepHLAPred. Five-fold cross-validation is to randomly split the dataset into five parts, of which four parts are used for training the model and the other is used for testing the model. The training and testing process is repeated five times to ensure that each is trained four times and tested only a time. As shown in Fig. 2, the DeepHLAPred achieved excellent performance, with AUC reaching 98.92%, 98.12%, 98.55%, 95.95%, and 93.84% on five datasets of HLA-G*01:01, HLA-G*01:03, HLA-G*01:04, HLA-E*01:01, and HLA-E*01:03, respectively. For intuitively contrasting the DeepHLAPred to the HLAncPred which is the latest method for non-classical HLA Class I binder prediction, we draw histograms of SN, SP, ACC, MCC, and AUC (Fig. 3). Except for the SN on the datasets HLA-G*01:04 and HLA-E*01:03, and the AUC on the datasets HLA-G*01:01 and HLA-E*01:01, the DeepHLAPred obviously outperformed the HLAncPred. The DeepHLAPred improved SN by 1.70%, SP by 1.02%, ACC by 1.37%, and MCC by 3.05% on the dataset HLA-G*01:01. The DeepHLAPred increased SN by 5.21%, SP by 2.22%, ACC by 3.72%, MCC by 6.83%, and AUC by 1.12% on the dataset HLA-G*01:03. The DeepHLAPred promoted SP by 2.43%, ACC by 0.48%, MCC by 0.79%, and AUC by 0.55% on the dataset HLA-G*01:04. The DeepHLAPred raised SN by 1.27%, SP by 3.92%, ACC by 2.79%, and MCC by 5.55% on the dataset HLA-E*01:01. The DeepHLAPred elevated SP by 8.61%, ACC by 2.35%, MCC by 3.31%, and AUC by 0.84% on the dataset HLA-E*01:03. We performed 5-fold cross validations 5 times and used T-test to compare difference between DeepHLAPred and the HLAncPred. As shown in Table 2, most metrics were significantly improved excluding AUC on the HLA-E*01:01, SN on the HLA-E*01:03, and SN on the HLA-G*01:04.

**Table 2:** The *P*-values by T-test

*P*-Value	SN	SP	ACC	MCC	AUC
HLA-E*01:01	**0.0048**	**0.0036**	**0.00002**	**0.00004**	0.0610
HLA-E*01:03	0.9318	**0.0000004**	**0.00002**	**0.00003**	**0.0014**
HLA-G*01:01	**0.00000002**	**0.0004**	**0.000001**	**0.000001**	**0.0002**
HLA-G*01:03	**0.00000007**	**0.011**	**0.000007**	**0.000008**	**0.0002**
HLA-G*01:04	0.7370	**0.0025**	**0.0098**	**0.0105**	**0.0056**

### Validation on the imbalanced category

**Fig. 4:**
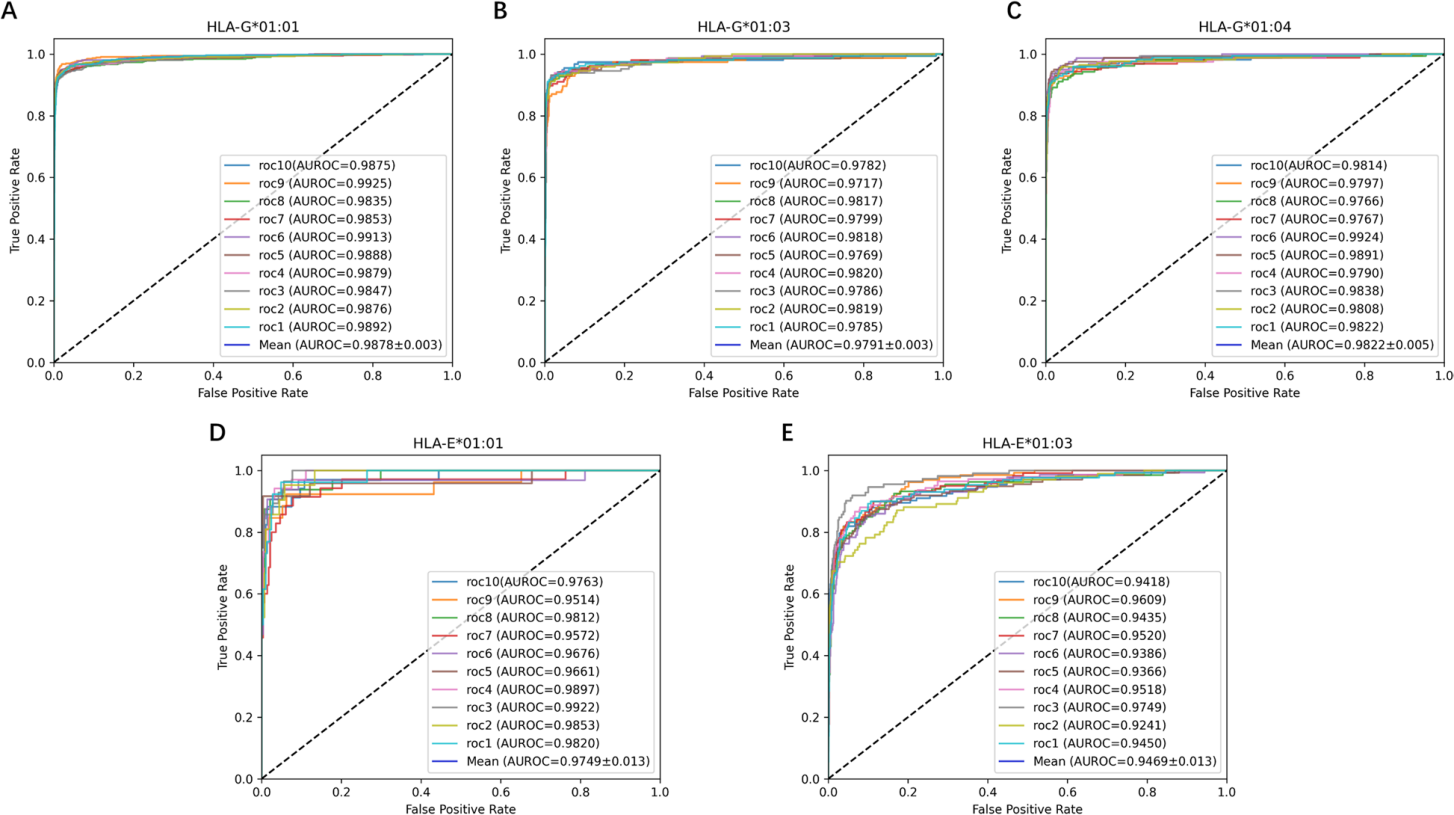
The ROC curves of 10-times shuffle validation on the imbalanced category

To further validate the effectiveness and efficiency of the DeepHLAPred, we amplified the numbers of negative samples ten times, which along with positive samples were called the imbalanced category (see the section Materials and methods). We shuffled samples in each dataset and randomly chose 10% samples for testing. We repeated this operation ten times. Figure 4 showed the ROC curves and the average ROC curves. The DeepHLAPred obtained the average AUC of 98.78% $\pm$ 0.003 on the HLA-G*01:01, 97.91% $\pm$ 0.003 on the HLA-G*01:03, 98.22% $\pm$ 0.005 on the HLA-G*01:04, 97.49% $\pm$ 0.013 on the HLA-E*01:01, and 94.69% $\pm$ 0.013 on the HLA-E*01:03 respectively. By contrast with Fig. 3, AUC was generally stable on the whole.

### Comparison with the state-of-the-art methods

It’s crucial to examine the performance of the DeepHLAPred on the independent datasets so as to objectively estimate its generalization ability. We retrieved 82 positive samples for HLA-E*01:01 and 67 positive ones for HLA-E*01:03 from the IEDB database [67], We randomly selected an equal number of negative samples from the imbalanced category, and none of these data were previously present in the training datasets. The positive along with negative samples constituted two independent datasets. We compared the DeepHLAPred with the state-of-the-art methods: HLAncPred ((https://webs.iiitd.edu.in/raghava/hlancpred) [6], MHCflurry 2.0 [21], NetMHCpan 4.1 (https://services.healthtech.dtu.dk/services/NetMHCpan-4.1/) [68]). As shown in the Table 3, DeepHLAPred demonstrated stable and excellent performance on the independent datasets. Although it was inferior to other three methods in terms of SP, DeepHLAPred exhibited greater stability in the prediction of different allele types, and it significantly outperformed MHCflurry 2.0 and NetMHCpan 4.1 in terms of SN, ACC, and MCC. Compared to HLAncPred, DeepHLAPred achieved a notable improvement on the HLA-E*01:01 dataset, increasing SN by 13.41%, ACC by 4.27%, and MCC by 6.03%. On the HLA-E*01:03 dataset, DeepHLAPred achieved performance comparable to HLAncPred, with a slight decreased SN by 1.5% but an increase of 1.49% of SP. Additionally, ACC and MCC were very close between the two methods.

**Table 3:** Comparisons with the state-of-the-art methods on independent datasets

Datasets	DeepHLAPred				HLAncPred				MHCflurry 2.0				NetMHCpan 4.1			
	SN	SP	ACC	MCC	SN	SP	ACC	MCC	SN	SP	ACC	MCC	SN	SP	ACC	MCC
HLA-E*01:01	0.7195	0.8780	0.7988	0.6052	0.5854	0.9268	0.7561	0.5449	0.2804	0.9634	0.6220	0.3339	0.4756	0.9390	0.7073	0.4679
HLA-E*01:03	0.9402	0.8507	0.8955	0.7942	0.9552	0.8358	0.8955	0.7967	0.1493	0.9552	0.5522	0.1765	0.3880	0.9851	0.6866	0.4651

### Discussion

Generally speaking, a single category of representation was inadequate to represent a protein sequence to full advantage. To validate this view, we experimented with single category of representation and their combination. As listed in Tables 4, 5, 6, 7 and 8, the INM performed best, followed by the EIIP, and the AAAF performed worst among the single category of representation. For example, the INM exceeded the AAAF by 31.52% ACC, the EIIP by 4.65% ACC on the dataset HLA-G*01:01. Difference in the performance between the EIIP and the INM was not too much. This indicated that the EIIP and INM better represent the peptide sequence. The combination of the AAAF, the INM and the EIIP reached the best ACC among the combination of any two and any single category of representation. This indicated that this combination enables complementation of different information.

**Table 4:** The performance of single representation and combinations on HLA-G*01:01

HLA-G*01:01	SN	SP	ACC	MCC	AUC
AAAF	0.6010	0.5382	0.5699	0.1397	0.5965
EIIP	0.8465	0.8304	0.8386	0.6774	0.9069
INM	0.9167	0.8529	0.8851	0.7720	0.9488
AAAF + EIIP	0.9102	0.8619	0.8859	0.7729	0.9532
AAAF + INM	0.9325	0.8504	0.8914	0.7856	0.9572
INM + EIIP	**0.9465**	0.9052	0.9253	0.8523	**0.9788**
INM + EIIP + AAAF	0.9407	**0.9145**	**0.9276**	**0.8557**	0.9762

**Table 5:** The performance of single representation and combinations on HLA-G*01:03

HLA-G*01:03	SN	SP	ACC	MCC	AUC
AAAF	0.5854	0.5440	0.5653	0.1299	0.5738
EIIP	0.8365	0.7814	0.8089	0.6190	0.8874
INM	0.8800	0.8298	0.8549	0.7107	0.9235
AAAF + EIIP	0.8525	0.8079	0.8296	0.6618	0.9116
AAAF + INM	0.9001	0.8083	0.8542	0.7115	0.9227
INM + EIIP	0.8990	**0.8685**	0.8835	0.7683	0.9537
INM + EIIP + AAAF	**0.9160**	0.8682	**0.8922**	**0.7852**	**0.9556**

**Table 6:** The performance of single representation and combinations on HLA-G*01:04

HLA-G*01:04	SN	SP	ACC	MCC	AUC
AAAF	0.5666	0.5646	0.5659	0.1315	0.5748
EIIP	0.8485	0.7964	0.8226	0.6462	0.9012
INM	0.8905	0.8470	0.8688	0.7389	0.9342
AAAF + EIIP	0.8630	0.8066	0.8350	0.6710	0.9079
AAAF + INM	0.9025	0.8419	0.8725	0.7471	0.9269
INM + EIIP	**0.9198**	0.8721	0.8960	0.7929	0.9547
INM + EIIP + AAAF	0.9137	0.**8875**	**0.9008**	**0.8022**	**0.9586**

**Table 7:** The performance of single representation and combinations on HLA-E*01:01.

HLA-E*01:01	SN	SP	ACC	MCC	AUC
AAAF	0.6595	0.6514	0.6559	0.3102	0.6815
EIIP	0.6848	0.7229	0.7041	0.4086	0.7781
INM	0.7977	0.7903	0.7917	0.5867	0.8534
AAAF + EIIP	0.7848	0.7251	0.7541	0.5099	0.8130
AAAF + INM	0.8447	0.7624	0.8029	0.6092	0.8430
INM + EIIP	**0.8458**	0.7807	**0.8144**	**0.6293**	**0.8512**
INM + EIIP + AAAF	0.8037	**0.7989**	0.7995	0.6022	0.8463

**Table 8:** The performance of single feature and combinations of features on HLA-E*01:03

HLA-E*01:03	SN	SP	ACC	MCC	AUC
AAAF	0.6150	0.5221	0.5689	0.1386	0.5867
EIIP	0.6870	0.6345	0.6605	0.3224	0.6979
INM	0.7059	0.6755	0.6946	0.3892	0.7392
AAAF + EIIP	0.7114	0.6560	0.6843	0.3687	0.7327
AAAF + INM	0.7217	0.6723	0.6969	0.3945	0.7481
INM + EIIP	0.7387	0.7109	0.7246	0.4498	0.7815
INM + EIIP + AAAF	**0.7451**	**0.7261**	**0.7358**	**0.4712**	**0.7901**

**Fig. 5:**
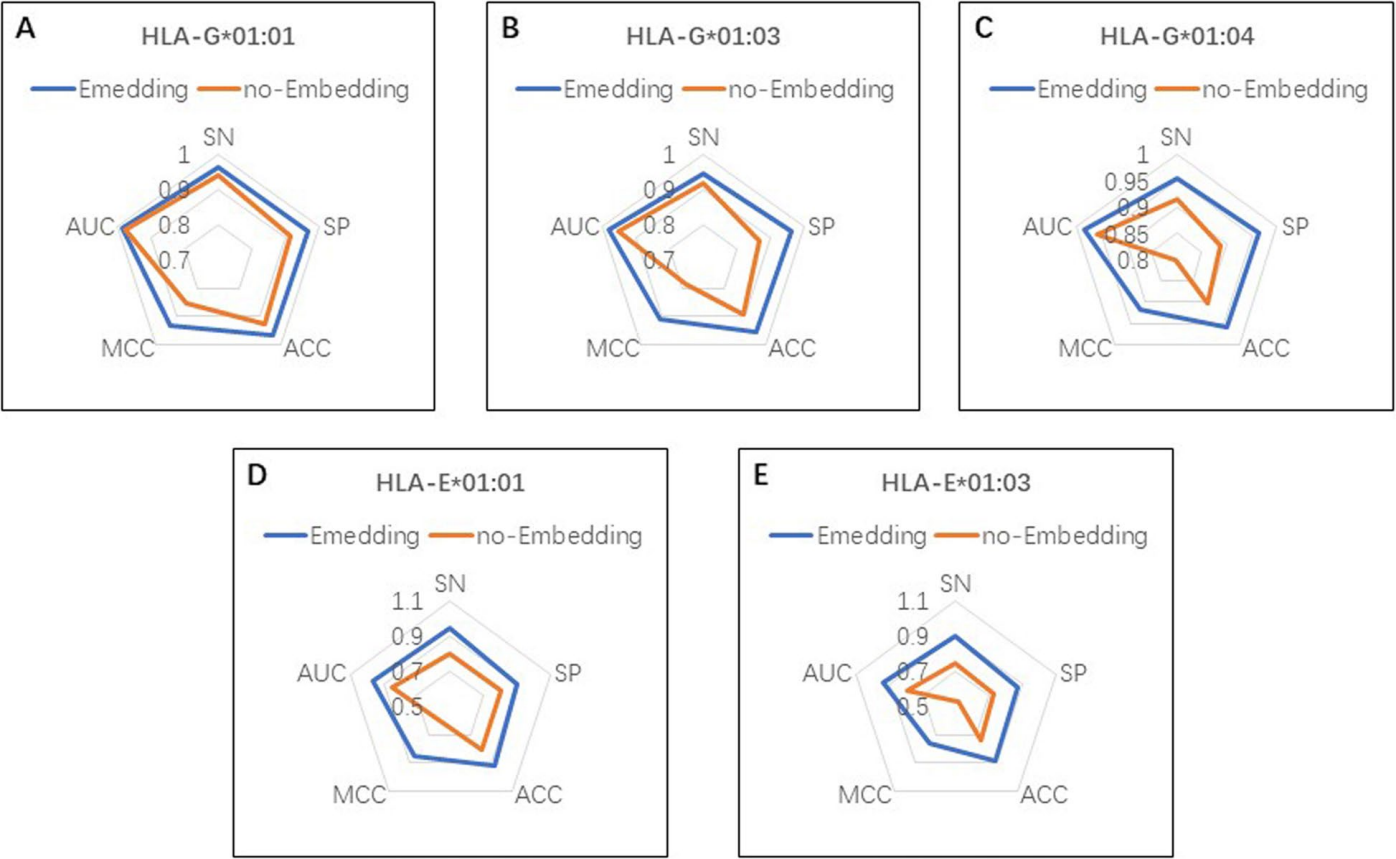
The radar chart of the performance Embedding layer

In the context of deep learning, the embedding layer is primarily intended to transform high dimensional discrete inputs into low dimensional continuous vectors. The embedding layer captures the correlation within the inputs, reduces computational complexity, and enhance the generalization ability. Therefore, the embedding layer is popularly used in the deep learning model. Figure 5 showed the performance of the DeepHLAPred with the embedding layer and without the embedding layer. The inclusion of the embedding layer significantly improved performance on each dataset. Take for example the dataset HLA-E*01:03, the DeepHLAPred without the Embedding layer obtained an SN of 74.51%, SP of 72.61%, ACC of 73.58%, MCC of 47.12%, and AUC of 79.01%, respectively, while the DeepHLAPred with the embedding layer, reached SN of 89.71%, SP of 86.56%, ACC of 88.12%, MCC of 76.31% and AUC of 93.84%, respectively. The inclusion of the embedding layer improved SN by 15.20%, SP by 13.95%, ACC by 14.54%, MCC by 29.19%, and AUC by 14.83%, respectively. Similar phenomenon was observed in the other datasets.

The DeepHLAPred comprised mainly two scales of CNN and Bi-LSTM. To demonstrate the superiority of the DeepHLAPred, we compared it with models with a single CNN, a single Bi-LSTM, a CNN followed by Bi-LSTM, two paralleling CNNs with different scales, and two paralleling Bi-LSTMs, their performance were shown in Tables 9, 10, 11, 12 and 13. The DeepHLAPred reached the better performance on the five datasets. We found that a single CNN model or single Bi-LSTM model is not as good as the CNN + Bi-LSTM combination. The above results demonstrated the soundness of the DeepHLAPred architecture.

**Fig. 6:**
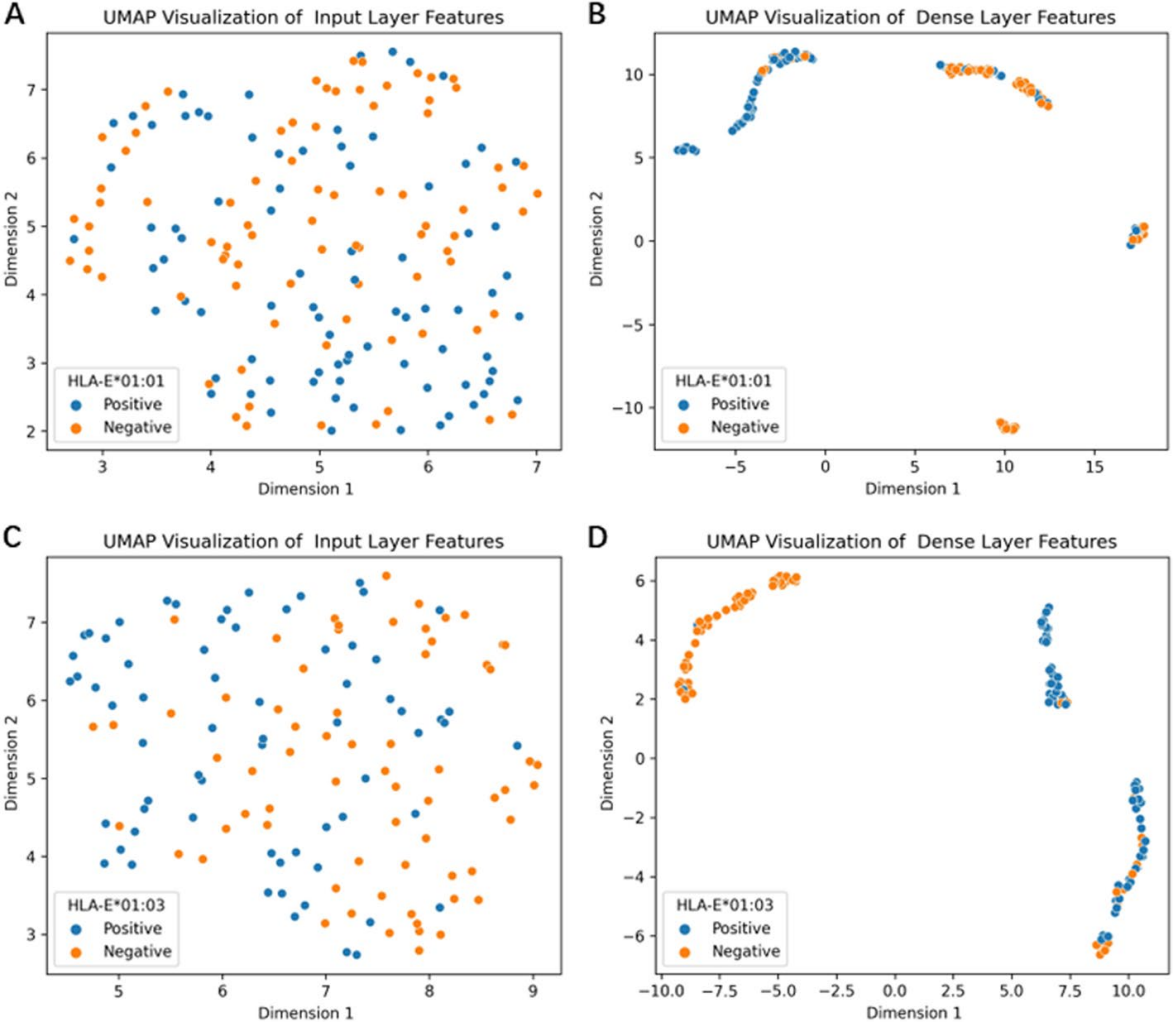
The Umap visualization for (**A**) initial representations, (**B**) learned representation on the HLA-E*01:01 dataset, (**C**) initial representations, (**D**) learned representation on the HLA-E*01:03 dataset. The learned representations refer to output of the first fully-connected layer

The discriminating ability of representations plays crucial roles in predictive performance. We used the Umap [38] to visualize the initial representations and the ones learned by the DeepHLAPred. As shown in Fig. 6, the DeepHLAPred remarkably improved the discriminating ability of representations.

**Table 9:** The performance of different modules on HLA-G*01:01 dataset

HLA-G*01:01	SN	SP	ACC	MCC	AUC
Model					
CNN	0.9467	0.9626	0.9548	0.9099	0.9872
Bi-LSTM	0.9482	0.9615	0.9550	0.9101	0.9863
CNN + Bi-LSTM (In series)	0.9533	0.9619	0.9577	0.9154	0.9873
CNN + CNN (In parallel)	0.9582	0.9593	0.9588	0.9176	0.9864
Bi-LSTM + Bi-LSTM (In parallel)	0.9529	0.9528	0.9529	0.9058	0.9861
DeepHLAPred	**0.9620**	**0.9685**	**0.9653**	**0.9305**	**0.9892**

**Table 10:** The performance of different modules on HLA-G*01:03 dataset

HLA-G*01:03	SN	SP	ACC	MCC	AUC
Model					
CNN	0.9372	0.9039	0.9208	0.8424	0.9726
Bi-LSTM	0.9257	0.9241	0.9248	0.8499	0.9716
CNN + Bi-LSTM (In series)	0.9281	0.9402	0.9341	0.8686	0.9786
CNN + CNN (In parallel)	0.9321	0.9333	0.9328	0.8656	0.9748
Bi-LSTM + Bi-LSTM (In parallel)	0.9309	0.9437	0.9374	0.8749	0.9703
DeepHLAPred	**0.9454**	**0.9626**	**0.9541**	**0.9083**	**0.9812**

**Table 11:** The performance of different modules on HLA-G*01:04 dataset

HLA-G*01:04	SN	SP	ACC	MCC	AUC
Model					
CNN	0.9264	0.9365	0.933	0.8627	0.9782
Bi-LSTM	0.9236	0.9234	0.9236	0.8475	0.9729
CNN + Bi-LSTM (In series)	0.9457	0.9394	0.9386	0.8858	0.9836
CNN + CNN (In parallel)	0.9310	0.9396	0.9354	0.8727	0.9802
Bi-LSTM + Bi-LSTM (In parallel)	0.9211	0.9434	0.9323	0.8657	0.9760
DeepHLAPred	**0.9545**	**0.9630**	**0.9587**	**0.9179**	**0.9855**

**Table 12:** The performance of different modules on HLA-E*01:01 dataset

HLA-E*01:01	SN	SP	ACC	MCC	AUC
Model					
CNN	0.8497	0.8512	0.8521	0.7050	0.9540
Bi-LSTM	0.8666	0.852	0.8588	0.7174	0.9209
CNN + Bi-LSTM (In series)	0.8475	0.8730	0.8629	0.7234	0.9356
CNN + CNN (In parallel)	0.9012	0.8588	0.8802	0.7605	0.9509
Bi-LSTM + Bi-LSTM (In parallel)	0.8721	0.8665	0.8690	0.7378	0.9391
DeepHLAPred	**0.9413**	**0.9013**	**0.9226**	**0.8455**	**0.9595**

**Table 13:** The performance of different modules on HLA-E*01:03 dataset

HLA-E*01:03	SN	SP	ACC	MCC	AUC
Model					
CNN	0.8507	0.8221	0.8377	0.6760	0.9130
Bi-LSTM	0.8448	0.8063	0.8259	0.6518	0.9053
CNN + Bi-LSTM (In series)	0.8905	0.8235	0.8575	0.7168	0.9287
CNN + CNN (In parallel)	0.8509	0.8464	0.8488	0.6974	0.9164
Bi-LSTM + Bi-LSTM (In parallel)	0.8511	0.8167	0.8346	0.6694	0.9031
DeepHLAPred	**0.8971**	**0.8656**	**0.8812**	**0.7631**	**0.9384**

### Information entropy analysis

We explored further potential sequence patterns of non-classical class-I HLA binding peptides from two perspectives: amino acid information entropy and positional information entropy. The position specific amino acid matrix was defined by:

10 $$\begin{array}{c}Z=\begin{pmatrix}\begin{array}{ccc}z_\text{1,1}&z_\text{1,2}&\cdots\\z_\text{2,1}&z_\text{2,2}&\cdots\\\vdots&\vdots&\vdots\end{array}&\begin{array}{c}z_{1,n}\\z_{2,n}\\\vdots\end{array}\\\begin{array}{ccc}z_\text{20,1}&z_\text{20,2}&\cdots\end{array}&z_{20,n}\end{pmatrix}\end{array}$$

where ${z}_{i,j}$ stood for the probability of the amino acid $i$ at the position $j$ and $n$ represented the length of the sequence. The position specific amino acid matrix was estimated in practice by calculating all the samples in the balanced datasets. The amino acid information entropy and the position information entropy were calculated as:

11 $$\begin{array}{c}A{P}^{i}={\sum\nolimits}_{j=1}^{n}-{Z}_{i,j}log\left({Z}_{i,j}\right)\end{array}$$

and

12 $$\begin{array}{c}{PP}^{j}={\sum\nolimits}_{i=1}^{20}-{Z}_{i,j}\text{log}\left({Z}_{i,j}\right)\end{array}$$

**Fig. 7:**
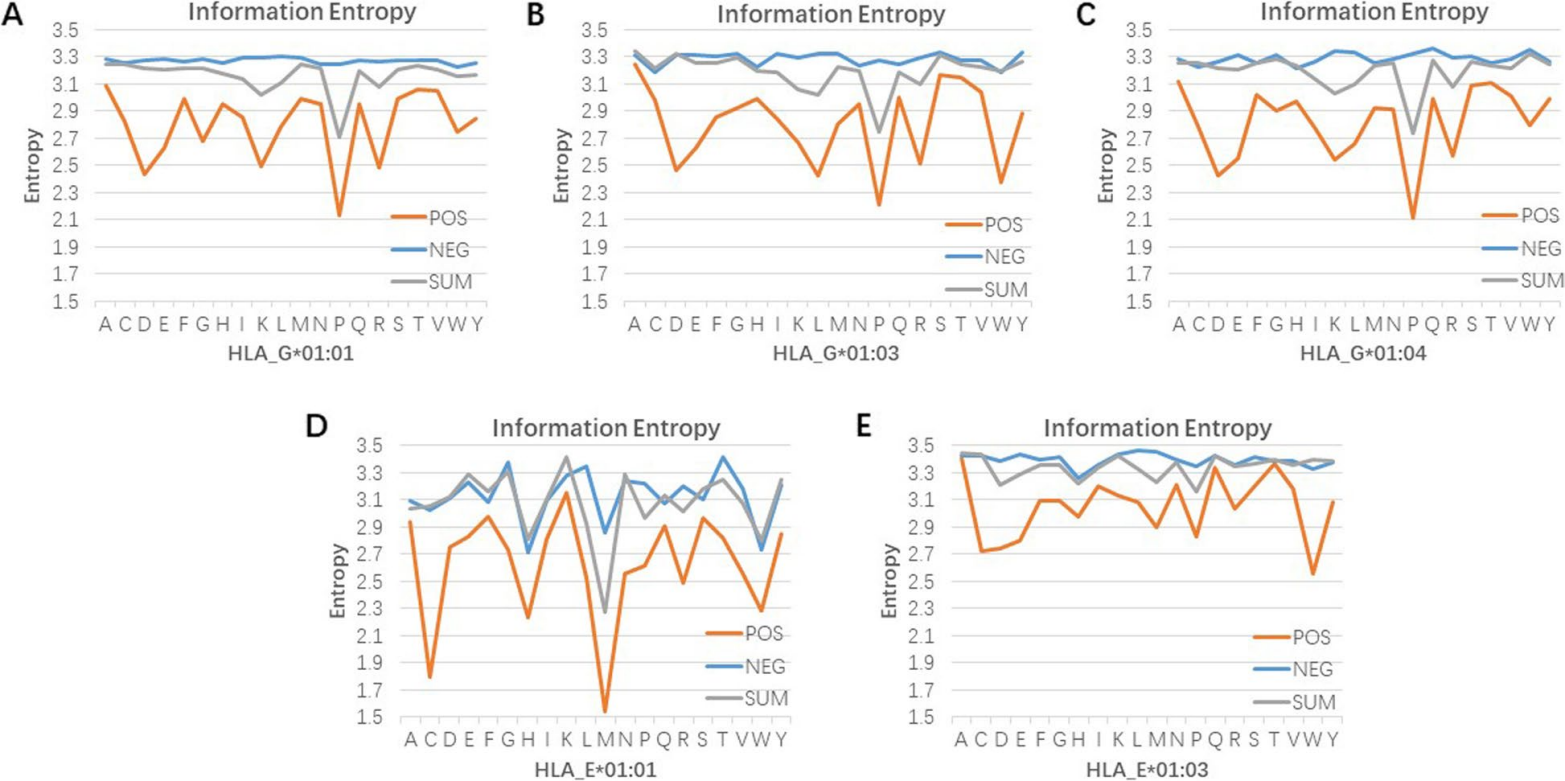
Amino acids information entropy. “POS”, “NEG”, and “SUM” represent positive samples, negative samples, and the total samples, respectively

**Fig. 8:**
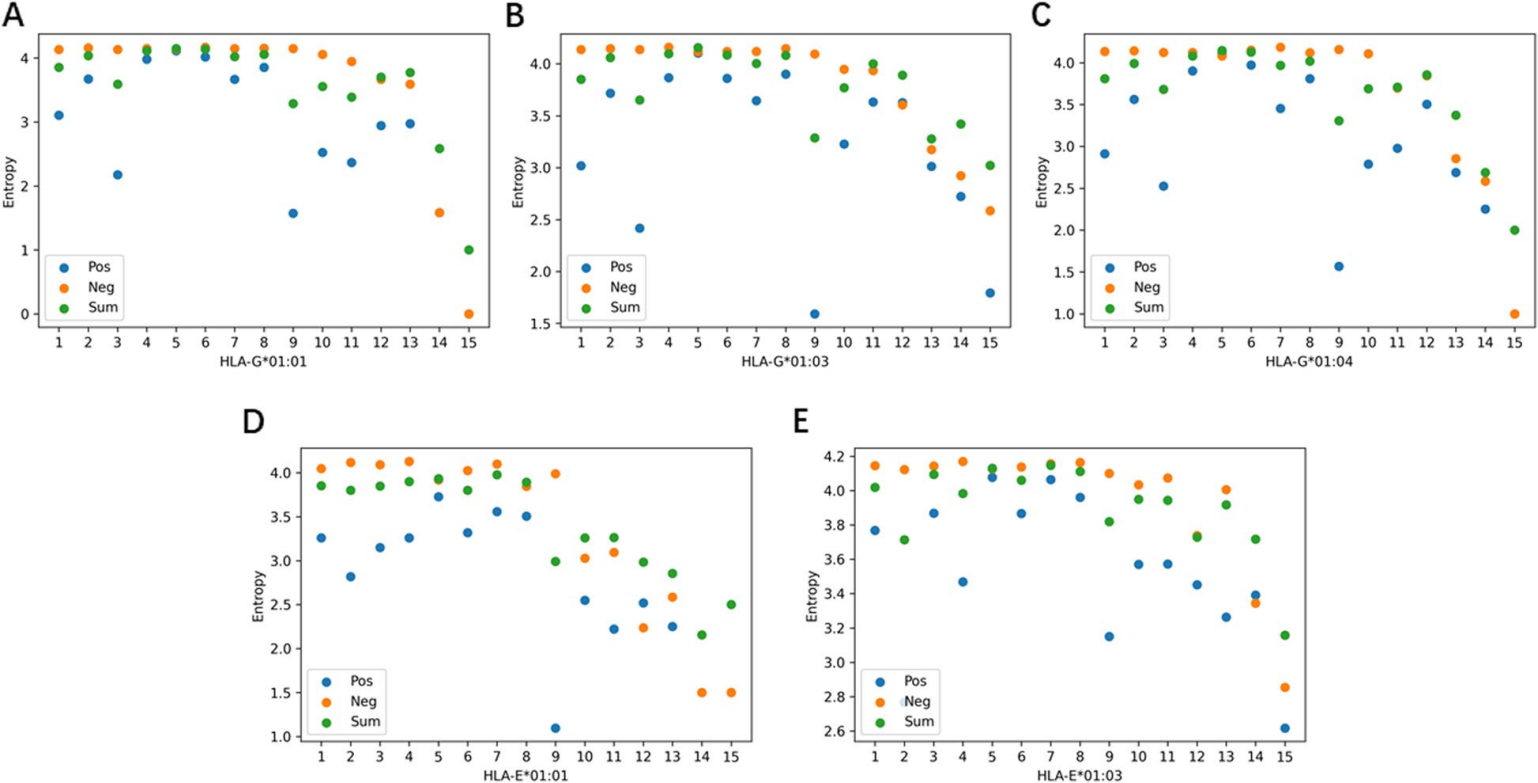
The position information entropy of non-classical HLA peptide sequences

The lower the information entropy was, the more certain the distribution of amino acid and position was. Figure 7 showed the amino acid information entropy on five balanced datasets. Evidently, HLA binding peptides generally have lower entropy values than the non-HLA binding peptides, indicating that the distribution of amino acid was not completely random. Amino acid information entropy exhibited specificity to the type of HLA binding peptides. The HLA-G binding peptides have lower value of amino acid information entropy at the Asparticacid (D) and Proline (P), while the HLA-E binding peptides have lower value at Cystine (C), Methionine (M), and Tryptophan (W). This implied that these amino acids were not distributed randomly. As shown in Fig. 8, we found that the positional information entropy of peptide sequences also was specific to type of HLA-binding peptides. Interestingly, positional information entropy at the 9-th position in the HLA-E*01:01, HLA-G*01:03, and HLA-G*01:04 were lower than others, indicating specificity of amino acid distribution at this position. These findings help us understand the sequence pattern of non-classical HLA I binding peptides [6, 21].

### Webserver

**Fig. 9:**
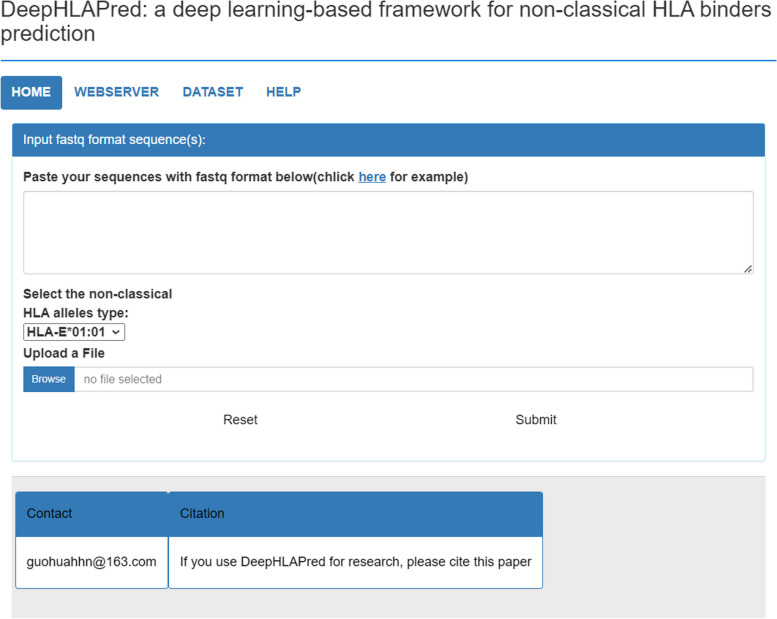
The web server page of the DeepHLAPred

To facilitate to predict non classical HLA class I binders, we developed a user-friendly webserver which is available at http:/www.biolscience.cn/DeepHLApred/. The webserver interface was shown in the Fig. 9. Users who utilize this webserver hardly require any prior knowledge about biology or deep learning. The only done is three steps. Firstly, users either input sequences in FASTA format into the inputting box or choose to upload a FASTA sequence file. Secondly, users select types of the non-classical HLA Class I allele which they want to predict. Finally, by clicking the submit button, users will get the prediction results on the webpage.

## Conclusion

HLA is closely related to the human immune system. Precisely identifying the HLA binding peptides is still challenging. We used three feature extraction methods, EIIP, AAAF, and INM to encode peptide sequences, and proposed a CNN and Bi-LSTM-based deep learning model (DeepHLAPred) for non-classical HLA Class I binder prediction. The DeepHLAPred was extensively tested by datasets of non-classical HLA I binder. It was well demonstrated that our method achieved state of the art performance on nearly all the datasets. The information entropy analysis implied the sequence pattern of non-classical binder to a certain extent. Though the DeepHLAPred demonstrated satisfactory performance in the prediction of non-classical HLA class I binding peptides. However, there still exists considerable room for improvement. In addition, the model interpretability need improving. In the future work, we shall focus on large language mode to improve prediction accuracy and interpretability.

### Supplementary Information

**Additional file 1:** **Supplementary Table 1.** The hyper-parameters of the DeepHLAPred. **Supplementary Table 2.** The performance on the HLA-G*01:01 dataset at different dropout rate. **Supplementary Table 3.** The performance on the HLA-G*01:03 dataset at different dropout rate. **Supplementary Table 4.** The performance on the HLA-G*01:04 dataset at different dropout rate. **Supplementary Table 5.** The performance on the HLA-E*01:01 dataset at different dropout rate. **Supplementary Table 6.** The performance on the HLA-E*01:03 dataset at different dropout rate. **Supplementary Table 7.** Comparison with state-of-the-art methods on five-fold cross-validation.

## Data Availability

The experimental data was available at https://github.com/tangxingyu0/DeepHLApred.
