# Supplementary material for: DeepHLAPred: a deep learning-based method for non-classical HLA binder prediction
Source: BMC Genomics. 2023 Nov 23;24:706. doi: 10.1186/s12864-023-09796-2 (PMC10666343; doi:10.1186/s12864-023-09796-2)
Supplement: Supplementary file 1 — Additional file 1: Supplementary Table 1. The hyper-parameters of the DeepHLAPred. Supplementary Table 2. The performance on the HLA-G*01:01 dataset at different dropout rate. Supplementary Table 3. The performance on the HLA-G*01:03 dataset at different dropout rate. Supplementary Table 4. The performance on the HLA-G*01:04 dataset at different dropout rate. Supplementary Table 5. The performance on the HLA-E*01:01 dataset at different dropout rate. Supplementary Table 6. The performance on the HLA-E*01:03 dataset at different dropout rate. Supplementary Table 7. Comparison with state-of-the-art methods on five-fold cross-validation. [file 12864_2023_9796_MOESM1_ESM.docx]

Supplementary Table 1 The hyper-parameters of the DeepHLAPred

| Layer | Hyper-Parameter | Value |
| --- | --- | --- |
| Embedding | Embedding dimensions | 45 |
| Conv Layer 1 | Number of kernels  Size of kernels | 32  10 |
| Conv Layer 2 | Number of kernels  Size of kernels | 32  8 |
| Maxpooling Layer 1 | Pool size | 2 |
| Maxpooling Layer 2 | Pool size | 2 |
| Activation 1 | Activation function | relu |
| Activation 2 | Activation function | relu |
| BatchNormalization 1 | Parameters | 128 |
| BatchNormalization 2 | Parameters | 128 |
| Dropout 1 | Dropout rate | 0.5 |
| Dropout 2 | Dropout rate | 0.5 |
| Bi-LSTM Layer 1 | Number of neurons | 16 |
| Bi-LSTM Layer 2 | Number of neurons | 16 |
| Dense 1 | Number of neurons  Activate function | 16  relu |
| Dense 2 | Number of neurons  Activate function | 1  sigmoid |

Supplementary Table 2 The performance on the HLA-G*01:01 dataset at different dropout rate.

| HLA-G*01:01  Dropout rate | SN | SP | ACC | MCC | AUC |
| --- | --- | --- | --- | --- | --- |
| 0.1 | 0.8046 | 0.9968 | 0.9793 | 0.8689 | 0.9896 |
| 0.3 | 0.8824 | 0.9956 | 0.9853 | 0.9090 | 0.9888 |
| 0.5 | 0.9241 | 0.9941 | 0.9877 | 0.9254 | 0.9925 |

Supplementary Table 3 The performance on the HLA-G*01:03 dataset at different dropout rate.

| HLA-G*01:03  Dropout rate | SN | SP | ACC | MCC | AUC |
| --- | --- | --- | --- | --- | --- |
| 0.1 | 0.9091 | 0.9773 | 0.9710 | 0.8395 | 0.9764 |
| 0.3 | 0.9156 | 0.9813 | 0.9752 | 0.8605 | 0.9808 |
| 0.5 | 0.9286 | 0.9820 | 0.9770 | 0.8713 | 0.9782 |

Supplementary Table 4 The performance on the HLA-G*01:04 dataset at different dropout rate.

| HLA-G*01:04  Dropout rate | SN | SP | ACC | MCC | AUC |
| --- | --- | --- | --- | --- | --- |
| 0.1 | 0.8742 | 0.9834 | 0.9737 | 0.8412 | 0.9761 |
| 0.3 | 0.8994 | 0.9779 | 0.9709 | 0.8319 | 0.9697 |
| 0.5 | 0.9245 | 0.9810 | 0.9759 | 0.8608 | 0.9767 |

Supplementary Table 5 The performance on the HLA-E*01:01 dataset at different dropout rate.

| HLA-E*01:01  Dropout rate | SN | SP | ACC | MCC | AUC |
| --- | --- | --- | --- | --- | --- |
| 0.1 | 0.7692 | 0.9791 | 0.9617 | 0.7483 | 0.9788 |
| 0.3 | 0.8077 | 0.9756 | 0.9617 | 0.7575 | 0.9841 |
| 0.5 | 0.9063 | 0.9858 | 0.9776 | 0.8800 | 0.9676 |

Supplementary Table 6 The performance on the HLA-E*01:03 dataset at different dropout rate.

| HLA-E*01:03  Dropout rate | SN | SP | ACC | MCC | AUC |
| --- | --- | --- | --- | --- | --- |
| 0.1 | 0.8125 | 0.9586 | 0.9468 | 0.6887 | 0.9613 |
| 0.3 | 0.8393 | 0.9633 | 0.9533 | 0.7235 | 0.9746 |
| 0.5 | 0.9018 | 0.9547 | 0.9504 | 0.7324 | 0.9749 |

Supplementary Table 7 Comparison with state-of-the-art methods on five-fold cross-validation

| Datasets | DeepHLAPred | | | | | HLAncPred | | | | |
| --- | --- | --- | --- | --- | --- | --- | --- | --- | --- | --- |
|  | SN | SP | ACC | MCC | AUC | SN | SP | ACC | MCC | AUC |
| HLA-G*01:01 | **0.9620** | **0.9685** | **0.9653** | **0.9305** | 0.9892 | 0.9450 | 0.9583 | 0.9516 | 0.9000 | 0.9900 |
| HLA-G*01:03 | **0.9454** | **0.9626** | **0.9541** | **0.9083** | **0.9812** | 0.8933 | 0.9404 | 0.9169 | 0.8400 | 0.9700 |
| HLA-G*01:04 | 0.9545 | **0.9630** | **0.9587** | **0.9179** | **0.9855** | 0.9691 | 0.9387 | 0.9539 | 0.9100 | 0.9800 |
| HLA-E*01:01 | **0.9413** | **0.9013** | **0.9226** | **0.8455** | 0.9595 | 0.9286 | 0.8621 | 0.8947 | 0.7900 | 0.9600 |
| HLA-E*01:03 | 0.8971 | **0.8656** | **0.8812** | **0.7631** | **0.9384** | 0.9206 | 0.7795 | 0.8577 | 0.7300 | 0.9300 |
